# The Ætiology of Cancer

**Published:** 1899-04-22

**Authors:** 


### THE ÆTIOLOGY OF CANCER.

The most important problems, however, in regard to cancer are those which are concerned with its ætiology and its surgical treatment. First, let us take the conclusions arrived at by Mr. Plim ner in his article on the Ætiology and Histology of Cancer. They are practically the same as those contained in his recent paper read before the Royal Society on which we commented a few weeks ago. The following, he thinks, may be regarded as a summary of our present knowledge on the subject: (*a*) That in cancers there are certain intracellular bodies (which may also be found rarely outside the cells) which are neither parts of the cell structure, nor any known degenerative change, and which are only found in cancer; and that they are only found at the periphery of the growing parts of a cancer, and not in the degenerative parts, and that these bodies have distinctive micro-chemical reactions. (*b*) That there are certain cancers, which occur rarely, in which these bodies are present in enormous numbers. (*c*) That by the use of appropriate means these bodies can be isolated and cultivated outside the body. (*d*) That these cultures, when introduced into certain animals, can cause death, with the production of tumours, so far of endothelial origin (with the one exception of the cornea, the epithelium of which when inoculated with these organisms, proliferates considerably, both on the surface, and deeply in various directions between the fibrous layers of the cornea); and that pure cultures can be made from these tumours which, if inoculated into suitable animals, will again produce similar tumour growths. As to the exact nature of these intracellular bodies there is much difference of opinion. Following Metschnikoff, Mr. Plimmer is content, provisionally, to call them protozoa, although he thinks it probable that they will ultimately be found to be related to the saccharomycetes. What, however, seems certain is that they are living organisms, and capable of multiplication.

[1] Hospital, April 1.

